# Trends and factors associated with thinness among late adolescent girls in Ethiopia: Multivariate decomposition and multilevel analysis

**DOI:** 10.3389/fnut.2022.933895

**Published:** 2022-08-18

**Authors:** Melkamu Aderajew Zemene, Netsanet Worku Mengistu, Solomon Gedlu Nigatu

**Affiliations:** ^1^Department of Public Health, College of Health Sciences, Debre Tabor University, Debre Tabor, Ethiopia; ^2^Department of Human Nutrition, College of Medicine and Health Sciences, Institute of Public Health, University of Gondar, Gondar, Ethiopia; ^3^Department of Epidemiology and Biostatistics, College of Medicine and Health Sciences, Institute of Public Health, University of Gondar, Gondar, Ethiopia

**Keywords:** thinness, adolescent girls, Ethiopia, multivariate decomposition, multilevel

## Abstract

**Background:**

Undernutrition among adolescent girls is still a major public health problem in low- and middle-income countries (LMICs). Even though the global prevalence of thinness among adolescent girls declined over time, it remains steady in LMICs including Ethiopia. Therefore, this study aimed to assess the trends and factors associated with thinness.

**Methods:**

A logit-based multivariate decomposition analysis for a non-linear response model was fitted to identify factors that contributed to the change in thinness over time. For the associated factors, a multilevel binary logistic regression model was employed. The intra-class correlation coefficient (ICC) and likelihood ratio (LR) test were used to assess the presence of the clustering effect, and deviance was used for model comparison. Statistical significance was declared at *p* < 0.05.

**Results:**

Thinness among late adolescent girls declined significantly from 34.4% (95% CI: 32.8%, 36.0%) in 2000 to 24.9% (95% CI: 23.4%, 26.5%) in 2016 with an annual average reduction rate of 1.73%. About 84% of the decrement in thinness was attributed to the change in the effect of the characteristics. The place of residence and marital status were significantly associated with a change in thinness due to the change in coefficients. The compositional changes in the age of the adolescents, religion, and types of toilet facilities were also significantly associated with the change in thinness. From the multilevel binary logistic regression, higher age of adolescents (AOR = 0.83; 95% CI: 0.77, 0.90), improved toilet facility (AOR = 0.45; 95% CI: 0.31, 0.65), middle wealth index (AOR = 1.45; 95% CI: 1.10, 1.90), and female head of the household (AOR = 0.77; 95% CI: 0.61, 0.98) were significantly associated at an individual level, whereas being from Somali (AOR = 2.14; 95% CI:1.76, 3.10) and SNNP region (AOR = 0.35; 95% CI: 0.18, 0.68), they had a statistically significant association with thinness at community level.

**Conclusion:**

Thinness among late adolescent girls declined substantially, but it remains a major public health concern. Nutritional interventions targeting thinness reduction among late adolescent girls should base on the identified factors. Age, residence, marital status, type of toilet facility, religion, wealth index, sex of head of the household, and region were all associated with thinness in this study.

## Introduction

Adolescence is a phase of the human lifecycle extending from 10 to 19 years of age. Adolescents within this age group are in a critical transition period from childhood to adulthood (1). It is further classified as; early adolescence (10–14 years) and late adolescence (15–19 years) (2). Globally, the adolescent population represents 1.8 billion, where the majority (90%) of them reside in low and middle-income countries (LMICs). Yet, adolescents have been largely neglected from the public health agenda in LMICs (3). The global prevalence of thinness among adolescent girls decreased from 10% by the year 2014 to 8.4% in 2018. This shows that thinness in this age group remained fairly steady over the past decade (4). There is a significant disparity in the prevalence of thinness across the globe. For instance, adolescence thinness in South Asia and Sub-Saharan Africa is higher with a magnitude ranging from 32–65% to 15–58%, respectively, (5) compared to Northern America, Europe and Oceania, Latin America, and the Caribbean (6, 7). The lowest mean BMI among adolescent girls was from South East Asia followed by East Africa (Ref). Particularly, the lowest age-standardized mean BMI in girls was 16.8 kg/m^2^ which is from Ethiopia (4, 8). Growth and development of the body brought a hormonal change in adolescents which in turn leads to a change in body composition, macro, and micronutrient requirements. More than half of the adult body weight is gained during adolescence (9).

Undernourished adolescent girls grow slowly and sometimes if they get pregnant, they may not even finish growing before their first pregnancy. When energy intake in adolescents is constrained due to pregnancy, they will start to use their calories for the privilege of reproductively valuable adipose tissue formation at the expense of investing in their growth (4). In times of adolescent pregnancy, there is increased competition for nutrients with the fetus and there is a high risk of the mother becoming thin and the fetus becoming stunting (10).

Previous studies conducted on adolescent thinness revealed that rural residence, early marriage, and subsequent pregnancy, low educational status, poor access to safe water and sanitation, lack of health services targeted for adolescents, and low utilization of family planning were factors significantly associated with thinness among adolescent girls (11–17).

Despite countries having a range of nutrition programs and strategies, there is an implementational problem leaving thinness among adolescent girls still a major public health problem in LMICs, including Ethiopia (18). Thinness has short- and long-term consequences in adolescents. It has an impact on physical growth, mental development, and poor birth outcome that adversely affects cognitive development and school achievements, lowers productive capacity, and increases the risk of infections (19).

When the undernourished mother becomes pregnant, the next generation may also suffer from malnutrition and poor health (20).

Even though there have been different local studies on the prevalence and factors associated with thinness among adolescent girls in Ethiopia, there is limited evidence on the trends of thinness and factors that contributed to the change in thinness over time.

Therefore, this study has aimed to assess trends, the factors that are either positively or negatively contributing to the change in thinness prevalence, and the individual and community-level factors associated with thinness among late adolescent girls in Ethiopia. Thus, findings from this study will help policymakers, program managers, scholars, and healthcare providers in evaluating and designing strategies targeting influential factors for improving nutritional status in adolescents.

## Materials and methods

### Study design, area, and period

Secondary data analysis was conducted based on the four consecutive Ethiopian Demographic and Health Survey (EDHS) datasets (EDHS 2000, EDHS 2005, EDHS 2011, and EDHS 2016). EDHS is a community-based cross-sectional study conducted every 5 years to generate updated health and health-related indicators.

### Source and study population

The source population was all adolescent girls aged (15–19 years) in Ethiopia at the time of the survey years 2000, 2005, 2011, and 2016 EDHS. All late adolescent girls aged 15–19 years in Ethiopia at the time of the survey years of 2000, 2005, 2011, and 2016 EDHS in the selected Enumeration Areas (EAs) were the study population.

EDHS uses a two-stage stratified cluster sampling technique. In the first stage, a sample of EAs was selected independently from each stratum with proportional allocation stratified by residence (urban and rural) and region. In the second stage, from the selected EAs, a fixed number of households was taken by systematic sampling in each survey year. From the selected households, measurements of weight and height were taken from children aged 0–59 months, women 15–49 years, and men 15–59 years. A total weighted sample of 11,783 adolescent girls was included in this study. Of which, 3,456, 1,516, 3,724, and 3,087 weighted adolescent girls were screened in the 2000, 2005, 2011, and 2016 survey years, respectively. The detailed sampling procedures are available in the full EDHS reports (11–14).

### Eligibility criteria

The study included adolescent girls aged 15–19 years who were in the selected enumeration areas (EAs) in Ethiopia’s Demographic and Health Survey 2000, 2005, 2011, and 2016. Moreover, the study excluded adolescent girls who were pregnant or who gave birth in the last 2 months preceding the date of the interview. Additionally, adolescent girls who were not weighed/measured and whose values for weight and height were not recorded were excluded.

### Variables and data collection procedure

The outcome variable was thinness taken as a binary response; 0 coded for “not thin” and 1 coded for “thin.” The independent variables considered for this study were from two sources; individual-level variables (socio-economic and socio-demographic related factors, environmental factors, behavioral related factors) and community-level factors. The data were accessed and downloaded from the webpage of the international DHS program after subscribing as an authorized user. From EDHS, 2000, 2005, 2011, and 2016, we used the women dataset (IR recode) for this study.

### Operational definitions

#### Thinness (total underweight)

WHO classifies thinness as mild, moderate, and severe when the adolescent BMI for age z-score is < -1SD, < -2SD, and < -3SD, respectively, as compared with the median value of the world health organization reference point (21). In this study, thinness included mild, moderate, and severe. This operational definition is made to make it in line with DHS reports, where the data were taken from.

#### Wealth index

It is a composite measure of a household’s cumulative living standard divided into five quantiles using the wealth quantile data derived from principal component analysis (14).

#### Community poverty

It is defined as the proportion of late adolescent girls who resided in poor or poorest households in the cluster categorized as 0 for “low” (0–0.16) and 1 for “high” (0.161–1).

#### Community mass media exposure

It is defined as the proportion of late adolescent girls who had mass media exposure within the cluster categorized as 0 for “poor” (0–0.6) and 1 for “good” (0.61–1).

#### Community adolescent girls’ literacy

It is defined as the proportion of late adolescent girls who attended primary, secondary, or higher education within the cluster categorized as 0 for “low” (0–0.84) and 1 for “high” (0.85–1).

### Data management and analysis

The data were extracted, edited, coded, and verified by using STATA version 16/MP software. The analysis was conducted by using STATA, Microsoft Excel, and WHO Anthro Plus software accordingly. The overall analysis in this study was carried out on weighted data to restore representativeness and complex sampling procures were also considered during the testing of statistical significance.

### Data analysis

#### Trends of thinness among late adolescent girls

First, a descriptive analysis was done to observe the trends with a 95% confidence interval (CI) of thinness among adolescent girls in the four survey years. Similarly, the proportion of thinness for each factor in all periods was explored. The trends were explored separately for the periods 2000–2005, 2005–2011, 2011–2016, and 2000–2016.

Then, a logit-based multivariate decomposition analysis for a non-linear response model was implemented to determine the extent to which factors contributed to the observed change in thinness prevalence among adolescent girls.

Initially, a *p*-value of less than 0.25 was used to select candidate variables for multivariate decomposition analysis. A *p*-value of less than 0.05 with 95% CI was used to declare statistical significance after fitting to multivariate decomposition analysis in the overall decomposition and factors that contribute to the endowment and coefficient components.

Multivariate decomposition analysis is a non-linear model used to split the difference in a distribution statistic between two groups, or its change over time, into various explanatory factors. This statistical approach uses the output from regression models to partition the components of a group difference in a statistic, such as a mean or proportion, into a component attributable to compositional differences between groups; differences in characteristics (endowments), and a component attributable to differences in the effects of characteristics (differences in coefficients). This analysis technique is equally applicable for partitioning change over time into components attributable to changing composition and changing effects (22).

The dependent variable is the function of the linear combination of predictors and regression coefficients.


Y=F⁢(X⁢β)

where Y is the N × 1 dependent variable vector, X is an N × K matrix of independent variables, and β is a K × 1 vector of coefficients. F (⋅) is any once-differentiable function mapping a linear combination of X(Xβ) to Y. The overall differences in components that reflect compositional differences between groups (endowments) and differences in the effects of characteristics (coefficients) between two groups A and B can be decomposed as:


YA-YB=F⁢(XA⁢β⁢A)-F⁢(XB⁢β⁢B)

Logit(Y)A-logit(Y)B=F(XβA)A-F(XβB)B

=F(XβA)A-F(XβB)B⏟E+F(XβB)A-F(XβB)B⏟C

The component labeled “E” refers to the part of the differential attributable to differences in endowments or characteristics, usually called the explained component or characteristics effect. The “C” component is the difference attributable to coefficients (behavioral change) usually called the unexplained component. We have chosen group A as the comparison group and group B as the reference group. Thus, E reflects a counterfactual comparison of the difference in outcome from group A’s perspective (i.e., the expected difference if group A were given group B’s distribution of covariates). C reflects the counterfactual comparison of the difference in outcome from group B’s perspective (i.e., the expected differences if group B were experienced in group A’s behavioral response to X).

In this study, we applied a decomposition analysis to account for changes in thinness among adolescent girls between 2000 and 2016. The model for decomposition analysis was: Logit (A)-Logit (B) = [β0A-β0B] +ΣβijA [XijA-XijB] +ΣxijB[βijA-βijB], (22)

where,

→ β0A is the intercept in the regression equation for EDHS 2016.
→ β0B is the intercept in the regression equation for EDHS 2000.
→ βijA is the coefficient of the jth category of the ith determinant for EDHS 2016.
→ βijB is the coefficient of the jth category of the ith determinant for EDHS 2000
→ XijA is the proportion of the jth category of the ith determinant for EDHS 2016.
→ XijB is the proportion of the jth category of the ith determinant for EDHS 2000.

#### Factors associated with thinness among late adolescent girls

Finally, due to the hierarchical nature of the DHS data where individuals are nested in the community, multilevel binary logistic regression analysis was employed to identify the factors associated with thinness among late adolescent girls. In this analysis, four models were fitted for EDHS 2016: (1) a null model (**model I**) with no exposure variables to check the variability of thinness in the cluster, (2) the second model (**model II**) containing individual-level variables, (3) the third model (**model III**) containing community-level variables, and (4) the fourth model (**model IV**) containing both individual and community-level variables with the outcome variable.

The fixed effects (a measure of association) were used to estimate the association between thinness and explanatory variables for both individual and community-level factors. Explanatory variables with a *p* < 0.25 were selected for multivariable analysis. In the multivariable analysis, adjusted odds ratio (AOR) with 95% confidence interval (CI) was reported and statistical significance was declared at a *p* < 0.05. Multicollinearity was checked using correlation coefficient and variance inflation factor where variables with correlation coefficient less than 0.8 and VIF less than 10% were considered for the analysis.

Random effects (a measure of variation) were estimated by intra-class correlation coefficient (ICC), median odds ratio (MOR), and proportional change in variance (PCV).

Intra-class correlation coefficient (ICC) was the proportion of total variance in the outcome variable that was attributed to the area level. ICC of greater than 5% in the null model was used as a cut-off point to say there was a clustering or dependence (23).


ICC=VA/(VA+VI),where⁢VA⁢is⁢the⁢area-level⁢variance⁢and⁢VI⁢corresponds⁢to⁢individual-levelvariance(VI=π/23=3.29).

Median odds ratio (MOR) was defined as the median value of the odds ratio between the area at highest risk and the area at lowest risk when randomly picking out two areas. MOR shows the median probability of being thin determined by resident area. It aims to translate the area-level variance into a widely used odds ratio that has a consistent and intuitive interpretation. It showed unexplained heterogeneity between clusters (23).


MOR=exp.[√(2×VA)×0.6745],or MOR⁢e0.95VA

The proportional change in variance (PCV) measures the total variation attributed to individual and community-level factors in the multilevel model.


$$\mathrm{PCV}=\left(\mathrm{VA}-\mathrm{VB}\right)/{\mathrm{VA}}^{*}\,100$$

where VA = variance of the initial model and VB = variance of the model with more terms. The model performance was assessed using deviance information criteria (DIC) to select the best-fitted.

## Result

### Background characteristics of the study population

In this study, a total weighted sample of 11,783 adolescent girls was included. They had a mean ± standard deviation (SD) of age of 16.8 ± 1.39 years. The proportion of urban adolescent girls increased from 22.3% in 2000 to 26.8% in 2011 and slightly decreased to 23.6% in 2016. Regarding educational status, about 60% of adolescent girls were not educated in 2000 but significantly decreased to 13.4% in 2016. Besides, the proportion of adolescent girls who had primary education increased from 27.2% in 2000 to 63.6% in 2016 (Table 1).

**TABLE 1:** Percentage distribution of characteristics of adolescent girls in 2000, 2005, 2011, and 2016 Ethiopian demographic and health surveys.

Variables	Category	Weighted frequency (%)			
		EDHS 2000 (*N* = 3,456)	EDHS 2005 (*N* = 1,516)	EDHS 2011 (*N* = 3,724)	EDHS 2016 (*N* = 3,087)
Age	15	876 (25.4)	307 (20.2)	968 (26)	663 (21.5)
	16	763 (22.1)	318 (21)	778 (20.9)	668 (21.6)
	17	618 (17.9)	267 (17.6)	591 (15.8)	581 (18.8)
	18	733 (21.2)	403 (26.5)	874 (23.5)	790 (25.6)
	19	466 (13.4)	221 (14.7)	513 (13.8)	385 (12.5)
Residence	Urban	771 (22.3)	327 (21.6)	997 (26.8)	730 (23.6)
	Rural	2,685 (77.7)	1,189 (78.4)	2,727 (73.2)	2,357 (76.4)
Region	Tigray	217 (6.3)	112 (7.4)	286 (7.7)	252 (8.1)
	Afar	31 (0.9)	11 (0.7)	28 (0.7)	25 (0.8)
	Amhara	789 (22.8)	351 (23.2)	1,062 (28.5)	737 (23.9)
	Oromia	1,465 (42.3)	549 (36.2)	1,380 (37.0)	1,119 (36.2)
	Somali	38 (1.1)	34 (2.2)	57 (1.5)	86 (2.8)
	Ben-Gumz	38 (1.1)	12 (0.8)	36 (0.9)	29 (0.9)
	SNNP	652 (18.9)	341 (22.5)	637 (17.1)	601 (19.5)
	Gambela	7 (0.2)	3 (0.2)	16 (0.4)	8 (0.3)
	Harari	8 (0.2)	5 (0.3)	9 (0.3)	6 (0.2)
	Addis Ababa	193 (5.6)	90 (6.0)	196 (5.3)	206 (6.7)
	Dire dawa	17 (0.5)	7 (0.5)	14 (0.4)	17 (0.5)
Marital status	Not married	2,827 (81.8)	1,248 (82.3)	3,175 (85.3)	2,660 (86.2)
	Married	629 (18.2)	268 (17.7)	549 (14.7)	427 (13.8)
Religion	Orthodox	1,760 (51.0)	774 (51.0)	1,875 (51.2)	1,337 (43.3)
	Muslim	1,011 (29.2)	406 (27.0)	972 (26.6)	948 (30.7)
	Protestant	512 (14.8)	287 (18.9)	739 (20.2)	760 (24.6)
	Others*	173 (5.0)	48 (3.1)	72 (1.9)	42 (1.4)
Educational status	No education	2,072 (60)	596 (39.3)	604 (16.2)	415 (13.4)
	1^0^	943 (27.2)	665 (43.9)	2,643 (71.0)	1,963 (63.6)
	2^0^ and above	442 (12.8)	255 (16.8)	477 (12.8)	709 (23.0)
Wealth index	Poorest	–	230 (15.2)	613 (16.5)	423 (13.7)
	Poorer	–	265 (17.4)	645 (17.3)	501 (16.2)
	Middle	–	284 (18.7)	634 (17.0)	579 (18.8)
	Richer	–	271 (18.0)	831 (22.3)	670 (21.7)
	Richest	–	466 (30.7)	1,001 (26.9)	913 (29.6)
Occupation status	Not working	1,535 (44.4)	1,073 (70.8)	1,882 (50.5)	1,807 (58.5)
	Working	1,921 (55.6)	443 (29.2)	1,842 (49.5)	1,280 (41.5)
Family size	<6	1,914 (55.4)	859 (56.6)	2,137 (57.4)	1,878 (60.8)
	=6	1,542 (44.6)	658 (43.4)	1,587 (42.6)	1,209 (39.2)
HH head	Male	2,660 (77.0)	1,150 (75.9)	2,691 (73.3)	2,293 (74.3)
	Female	796 (23.0)	366 (24.1)	1,033 (27.7)	794 (25.7)
Age of HH head	<46	1,770 (51.2)	699 (46.1)	1,867 (50.1)	1,423 (46.1)
	=46	1,686 (48.8)	817 (53.9)	1,857 (49.9)	1,664 (53.9)
Toilet type	Unimproved	2,624 (76)	1,321 (87.1)	3,027 (81.3)	2,578 (83.5)
	Improved	832 (24)	195 (12.9)	697 (18.7)	509 (16.5)
Drinking water source	Unimproved	2,526 (73)	1,084 (71.5)	2,521 (67.7)	1,465 (47.5)
	Improved	930 (27)	432 (28.5)	1,203 (32.3)	1,622 (52.5)
Ever drink alcohol	No	–	–	2,193 (58.9)	2,111 (68.4)
	Yes	–	–	1,531 (41.1)	976 (31.6)
Ever chew chat	No	–	–	3,528 (94.7)	2,865 (92.8)
	Yes	–	–	196 (5.3)	222 (7.2)
Media exposure	No	1,980 (57)	623 (41.1)	839 (22.5)	1,461 (47.3)
	Yes	1,476 (43)	893 (58.9)	2,885 (77.5)	1,626 (52.7)
Anemia status	Anemic	–	333 (21.9)	475 (12.9)	590 (19.5)
	Normal	–	1,183 (78.1)	3,183 (87.1)	2,438 (80.5)

*Catholic/traditional/other. HH, household.

### Trends in thinness among late adolescent girls in the four survey years

**FIGURE 1:**
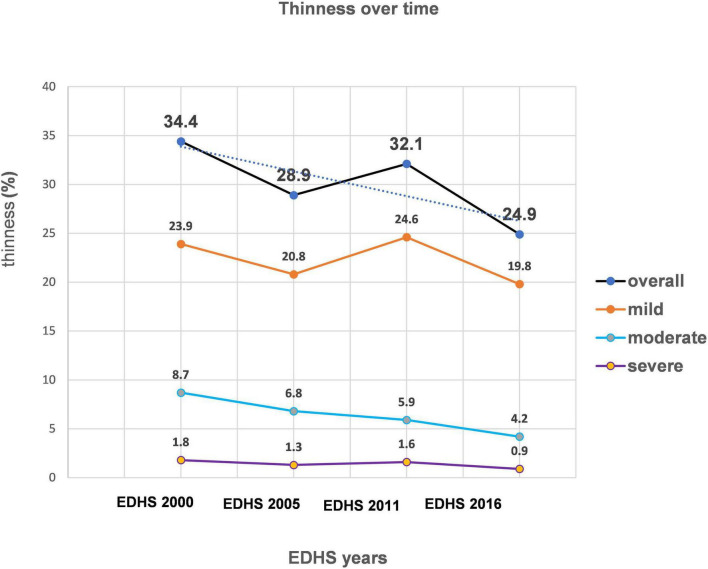
Trends of thinness among late adolescent girls in Ethiopia from EDHS 2000 to 2016.

The trend period was divided into four phases 2000–2005, 2005–2011, 2011–2016, and 2000–2016. In the overall trend, thinness among late adolescent girls has shown a significant decrement of 9.5% changing from 34.4% (32.8, 36.0) to 24.9% (23.4, 26.5) in the period 2000–2016 with an annual average reduction rate of 1.73%. The prevalence of mild, moderate, and severe thin late adolescent girls in EDHS 2000 was 23.9, 8.7, and 1.8% which declined to 19.8, 4.2, and 0.9% in EDHS 2016, respectively (Figure 1), indicating 50% or more reduction in moderate and severe forms of thinness within the 16 years of the data collection period.

### Trends of thinness in adolescent girls’ characteristics

The trend in thinness among late adolescent girls over the study period (2000–2016) varied in terms of different factors. For example, among rural residents, the overall change in thinness was 11.6%. The largest decrement was in the third phase (2011–2016) with an 8.1%point change, while the lowest was 3.4 in the second phase (2005–2011). Regarding marital status, adolescent girls who were not married showed a 7.8% point decrement in thinness in the period from 2011 to 2016 (Table 2).

**TABLE 2:** Trends in thinness among late adolescent girls in Ethiopia by the selected characteristics of 2000, 2005, 2011, and 2016 Ethiopian demographic and health surveys in percent (%).

Variables	2000 *N* = 3,456	2005 *N* = 1,516	2011 *N* = 3,724	2016 *N* = 3,087	Point difference in thinness rate			
					2005–2000	2011–2005	2016–2011	2016–2000
**Residence**								
Urban	20.6	19.5	24.4	18.9	−1.1	4.9	−5.5	−1.7
Rural	38.4	31.5	34.9	26.8	−6.9	3.4	−8.1	−11.6
**Marital status**								
Not married	35.7	29.7	32.2	24.4	−6	2.5	−7.8	−11.3
Married	28.6	25.4	31.3	28.5	−3.2	5.9	−2.8	−0.1
**Religion**								
Orthodox	34.2	29.6	37.2	28.7	−4.6	7.6	−8.5	−5.5
Muslim	36.5	29.9	29.1	26.4	−6.6	−0.8	−2.7	−10.1
Protestant	29.3	26.2	23.8	17.0	−3.1	−2.4	−6.8	−12.3
Others*	39.3	24.4	20.3	15.7	−14.9	−4.1	−4.6	−23.6
**Educational status**								
No education	35.9	28.0	25.3	26.6	−7.9	−2.7	1.3	−9.3
1^0^	36.7	31.6	35.2	25.8	−5.1	3.6	−9.4	−10.9
2^0^ and above	22.4	24.0	23.2	21.6	1.6	−0.8	−1.6	−0.8
**Wealth index**								
Poorest	–	36.0	39.1	32.1	–	3.1	−7	–
Poorer	–	28.1	39.5	21.6	–	11.4	−17.9	–
Middle	–	36.6	30.9	32.7	–	−5.7	1.8	–
Richer	–	32.5	32.6	22.7	–	0.1	−9.9	–
Richest	–	19.1	23.3	20.1	–	4.2	−3.2	–
**Occupation**								
Not working	36.9	29.9	32.4	26.1	−7	2.5	−6.3	−10.8
Working	32.4	26.6	31.8	23.3	−5.8	5.2	−8.5	−9.1
**Family size**								
<6	32.9	26.6	29.5	23.8	−6.3	2.9	−5.7	−9.1
=6	36.2	32.0	35.6	26.7	−4.2	3.6	−8.9	−9.5
**HHhead**								
Male	35.7	29.1	33.8	26.3	−6.6	4.7	−7.5	−9.4
Female	30.2	28.1	27.7	21.0	−2.1	−0.4	−6.7	−9.2
**Toilet type**								
Unimproved	37.8	30.5	32.9	26.5	−7.3	2.4	−6.4	−11.3
Improved	23.7	18.4	28.5	17.2	−5.3	10.1	−11.3	−6.5
**Water source**								
Unimproved	37.4	32.6	37.3	26.1	−4.8	4.7	−11.2	−11.3
Improved	33.3	27.4	29.6	23.6	−5.9	2.2	−6	−9.7
**Media exposure**								
No	38.1	34.7	34.7	27.7	−3.4	0	−7	−10.4
Yes	29.5	24.8	31.3	22.5	−4.7	6.5	−8.8	−7
**Anemia status**								
Anemic	–	29.5	34.4	24.9	–	4.9	−9.5	–
Normal	–	28.7	31.8	25.1	–	3.1	−6.7	–
Overall	34.4	28.9	32.1	24.9	−5.5	3.2	−7.2	−9.5

*Catholic/traditional/other. HH, household.

Based on region, Benishangul-Gumuz showed the largest decrement of 32.2% change in thinness in the period 2000–2016 Figure 2.

**FIGURE 2:**
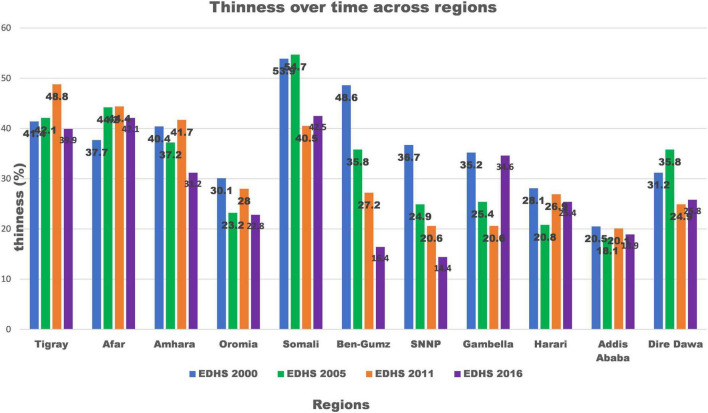
Trends of thinness among late adolescent girls over time across regions of Ethiopia from 2000 to 2016.

### Factors associated with a change in thinness

#### Decomposition analysis

##### Differences due to characteristics (endowments)

After controlling the role of changes due to coefficients, 16% of the decrement in thinness among late adolescent girls in Ethiopia in the last one and a half-decade was due to differences in characteristics (endowments). Among the compositional factors; age, religion, and types of toilet had a statistically significant contribution to the change in thinness. Thus, as compared to 15 years old, there was an increase in the proportion of adolescent girls who were 17 and 18 years old in the study period positively attributed to the change in thinness. But the decrement in the composition of 19-years-old adolescent girls in the sample showed a significant impact on the overall change in thinness (Table 3).

**TABLE 3:** Detail decomposition of the change in thinness among late adolescent girls in Ethiopia, 2000–2016.

Variables	Differences due to characteristics (E)		Difference due to coefficients (C)	
	Coeff (95% CI)	Pct (%)	Coeff (95% CI)	Pct (%)
Age (years)				
15	1	1	1	1
16	0.00029 (−0.000044, 0.00006)	−0.33134	0.00415 (−0.01637, 0.02469)	−4.4428
17	−0.00087 (−0.00159, −0.00014)*	0.92801	0.00956 (−0.00883, 0.02796)	−10.223
18	−0.00492 (−0.0084, −0.00145)**	5.2616	0.00694 (−0.01353, 0.02742)	−7.4171
19	0.00121 (0.00023, 0.0022)*	−1.288	0.00964 (−0.00661, 0.02588)	−10.294
Residence				
Urban	1	1	1	1
Rural	−0.00029 (−0.00130, 0.00070)	0.31809	−0.09425 (−0.18726, −0.00125)*	100.68
Marital status				
Married	1	1	1	1
Not married	−0.00221 (−0.00557, 0.00115)	2.3633	−0.10228 (−0.18102, −0.02354)*	109.26
HH head				
Male	1	1	1	1
Female	−0.00117 (−0.00265, 0.00030)	1.2574	−0.00082 (−0.01917, 0.01753)	0.8769
Family size				
<6	1	1	1	1
=6	−0.00097 (−0.00362, 0.00168)	1.0374	0.00897 (−0.02201, 0.03996)	−9.5887
Religion				
Orthodox	1	1	1	1
Muslim	−0.00068 (−0.00152, 0.00016)	0.7292	−0.01584 (−0.03746, 0.00577)	16.924
Protestant	−0.01504 (−0.0221, −0.0079)***	16.075	−0.01303 (−0.02697, 0.00090)	13.922
Others^$^	0.00664 (−0.00709, 0.02037)	−7.0947	−0.01048 (−0.02787, 0.00690)	11.197
Toilet type				
Unimproved	1	1	1	1
Improved	0.00669 (0.00043, 0.01296) *	−7.1554	−0.01117 (−0.03584, 0.01349)	11.939
Media exposure				
No	1	1	1	1
Yes	−0.00369 (−0.01073, 0.00333)	3.9515	−0.00549 (−0.03322, 0.02222)	5.8747
Constants			0.13558 (−0.02601, 0.29718)	−144.83
Overall	−0.015092 (−0.02869, −0.0015)*	16.121	−0.07852 (−0.11172, −0.0453)***	83.879

^$^Catholic/traditional/other. *p < 0.05, **p < 0.01, ***p < 0.001. HH, household; 1, reference; CI, confidence interval. The bold values represent the overall values.

Regarding religion, a result of an increase in the proportion of protestant followers from 14.8% in 2000 to 24.6% in 2016 had an important compositional contribution of 16% in the change in thinness. However, the proportion of adolescent girls from households that had improved toilets declined from 24.0% in 2000 to 16.5% in 2016. This change in the composition of the survey population resulted in a significant negative impact on the decrement of thinness (Table 3).

##### Difference due to the effect of coefficients

Controlling the role of changes in compositional characteristics, 84% of decrement in thinness among late adolescent girls in Ethiopia in the study period was attributed to change in the effect of the characteristics (coefficient). Residence and marital status were the most important factors that had a statistically significant contribution to the observed change in thinness (Table 3).

By keeping all the compositional change factors controlled, thinness significantly declined due to behavioral changes toward nutrition among rural residents during the study period. Furthermore, controlling all the compositional change factors, late adolescent girls who were not married had significantly contributed to the change in thinness as compared with those who were married as shown below (Table 3).

In the multivariate decomposition model fitted above, it should be noted that the intercept (0.13558) accounts for -144.83% of the overall decrement in thinness. This suggested that the model fit had some limitations in explaining the change in thinness between 2000 and 2016. Therefore, to reduce thinness, other factors could exist in nutrition programs and interventions targeting late adolescent girls.

### Multilevel logistic regression analysis

#### Random effect and model comparison results

As shown in Table 4, the intra-class correlation coefficient (ICC) in the null model was 0.16, which means about 16% of the variations of thinness among late adolescent girls were attributed to the difference at cluster level or community-level factors. This suggests the need for multilevel logistic regression rather than the standard flat model.

**TABLE 4:** Random effect and model fit statistics for thinness among late adolescent girls in Ethiopia by multilevel logistic regression analysis, EDHS 2016.

Parameters	Model I (Null)	Model II	Model III	Model IV
Coefficient variance	0.62	0.57	0.41	0.46
ICC (%)	16%	14.8%	11%	12%
MOR (CI)	2.12 (1.87, 2.46)	2.05 (1.78, 2.4)	1.84 (1.6, 2.1)	1.89 (1.68,2.2)
PCV (%)	Reff.	8%	34%	25.8%
**Model fitness**				
DIC (−2LL)	3,373	3,255	3,285	3,201
AIC	3,377	3,289	3,315	3,258
BIC	3,389	3,392	3,406	3,427

ICC, intra-class correlation coefficient; MOR, median odds ratio; PCV, proportional change in variance; DIC, deviance information criteria; AIC, Akaike’s information criterion; BIC, Bayesian information criterion; Model I is the null model, a baseline model without any independent variable. Model II is adjusted for individual-level factors. Model III is adjusted for community-level factors. Model IV is the full model adjusted for both individual and community-level factors.

The median odds ratio (MOR) value of 2.12 (1.87, 2.46) in the null model also revealed that there was unexplained heterogeneity among clusters since MOR was 2.12 times more than the reference (MOR = 1). The unexplained community variation in thinness declined to MOR of 1.89 (1.68, 2.2) when the model was adjusted to both individual and community-level factors in the final model. This showed that after all factors were considered, the effect of clustering was still statistically significant in the full model.

After adjusting for individual and community-level factors in the full model, the proportional change in variance (PCV) revealed that about 26% of variations of thinness among late adolescent girls were attributed to both individual and community-level factors in the multilevel model.

Regarding model comparison/fitness statistics, we used deviance information criteria (DIC). Then, the model with the lowest deviance information criteria (DIC), that is the one that had the highest likelihood ratio value (**model IV**) was the best-fitted model (Table 4).

#### Fixed effect results; multilevel logistic regression analysis

In the bi-variable analysis, variables like the source of drinking water, educational status, history of chat chewing, anemia status, and community-level literacy had a *p*-value of > 0.2. So, they were omitted from further analysis.

From the final model (model IV) results, the age of the respondent, sex of head of the household, age of head of the household, types of toilets, wealth index, and region had a statistically significant association with thinness among late adolescent girls.

But all the remaining independent individual and community-level variables had no statistically significant association with the outcome variable at a *p*-value of 0.05.

#### Individual-level factors

As shown in Table 5, for a 1-year increase in ages of late adolescent girls, the odds of being thin decreased by 17% (AOR = 0.83; 95% CI: 0.77,0.90), given that all the other variables held constant. Late adolescent girls from female-headed households were 23% (AOR = 0.77; 95% CI: 0.61, 0.98) less likely to be thin as compared to male-headed households keeping all other variables constant.

**TABLE 5:** Factors associated with thinness among late adolescent girls in Ethiopia by multilevel logistic regression analysis, EDHS 2016.

Variables	Model II AOR (95% CI)	Model III AOR (95% CI)	Model IV AOR (95% CI)
Individual-level factors			
Age of adolescents	0.84 (0.78, 0.90)***	–	0.83 (0.77, 0.90)***
Household head sex			
Male	1	–	1
Female	0.80 (0.63, 1.02)	–	0.77 (0.61, 0.98)*
Ever drink alcohol			
No	1	–	1
Yes	1.06 (0.79, 1.42)	–	0.95 (0.70, 1.28)
Occupation status			
Not working	1	–	1
Working	0.91 (0.69, 1.18)	–	0.87 (0.67, 1.15)
Family size			
Below 6	1	–	1
Six and above	1.19 (0.96, 1.46)	–	1.15 (0.93, 1.43)
Marital status			
Married	1	–	1
Not married	0.80 (0.59, 1.09)	–	0.82 (0.60, 1.11)
Religion			
Orthodox	1	–	1
Muslim	0.82 (0.59, 1.13)	–	0.87 (0.60, 1.28)
Protestant	0.43 (0.30, 0.62)***	–	0.71 (0.47, 1.06)
Others^#^	0.34 (0.11, 1.05)	–	055 (0.18, 1.71)
Media exposure			
No	1	–	1
Yes	0.84 (0.68, 1.03)	–	0.82 (0.65, 1.03)
Age of household head	0.97 (0.96, 0.99)**	–	0.96 (0.95, 1.07)
Toilet facility			
Unimproved	1	–	1
Improved	0.52 (0.38, 0.72)***	–	0.45 (0.31, 0.65)***
Wealth index			
Poor	0.97 (0.75, 1.26)	–	0.84 (0.62, 1.13)
Middle	1.46 (1.12, 1.89)**	–	1.45 (1.10,1.90) **
Rich	1	–	1
Community-level factors			
Residence	–		
Urban	–	1	1
Rural	–	1.76 (1.20, 2.57)**	1.18 (0.77, 1.80)
Region			
Addis Ababa	–	1	1
Tigray	–	2.08 (1.16, 3,72)*	1.61 (0.86, 2.98)
Afar	–	2.31 (0.86, 6.21)	1.83 (0.64, 519)
Amhara	–	1.39 (0.80, 2.41)	0.92 (0.51, 1.67)
Oromia	–	0.81 (0.47, 1.41)	0.60 (0.33, 1.08)
Somali	–	2.33 (1.14, 4.73)*	2.14 (1.0, 4.10) *
Benishangul-Gumz	–	0.53 (0.17, 1.68)	0.36 (0.11, 1.18)
SNNP	–	0.44 (0.25, 0.80)**	0.35 (0.18, 0.68) **
Gambelia	–	1.87 (0.39, 8.90)	1.36 (0.27, 6.85)
Harari	–	1.22 (0.17, 8.41)	0.92 (0.12, 6.70)
Dire Da’wa	–	1.36 (0.39, 4.68)	1.33 (0.37, 4.81)
Community media exposure			
Low	–	1	1
High	–	0.99 (073, 1.33)	1.11 (0.80, 1.55)
Community poverty			
Low	–	1	1
High	–	1.02 (0.75, 1.35)	1.02 (0.73, 1.42)

^#^Catholic/traditional/other; *p < 0.05; **p < 0.01; ***p < 0.001 1 = reference; CI, confidence interval. Model I is an empty model. Model II is adjusted for individual-level factors. Model III is adjusted for community-level factors. Model Iv is the full model adjusted for both individual and community-level factors.

The odds of being thin was 2.2 times (AOR = 0.45; 95% CI: 0.31, 0.65) higher for late adolescent girls from households that had unimproved toilets as compared to households that had improved toilet keeping other factors constant. Based on EDHS 2016 household wealth index classification, late adolescent girls from households with a middle wealth index had 45% higher odds of being thin as compared to late adolescent girls from the rich wealth index (AOR = 1.45; 95% CI: 1.10,1.90) assuming all other variables keep constant.

#### Community-level factors

As shown in Table 5, the region showed a statistically significant association (*p* < 0.05) with thinness among late adolescent girls in Ethiopia from EDHS 2016. The odds of being thin were 2.14 (AOR = 2.14; 95% CI: 1.0, 4.10) times higher for late adolescent girls from the Somali region as compared to their counterparts from Addis Ababa, keeping other factors constant. Moreover, late adolescent girls who were from South Nations Nationalities and people of Ethiopia region had 65% (AOR = 0.35; 95% CI: 0.18, 0.68) fewer odds of being thin as compared to late adolescents from Addis Ababa, held other variables constant (Table 5).

## Discussion

In this study, thinness among late adolescent girls has shown a significant decrement of 9.5% point change from 34.4% (32.8, 36.0) in 2000 to 24.9% (23.4, 26.5) in 2016. This could be attributed to the progress in the nutritional status of women of reproductive age and adolescent girls in the national nutrition program. Besides, the school health nutrition strategy and program in some parts of Ethiopia might be attributed to the observed change in thinness. Even though thinness in the study period showed a significant decrement, based on World Health Organization Nutrition Landscape Information System (NLIS) cut-off, it is still in the range of a high public health problem (24).

The multivariate decomposition analysis revealed that the contribution of behavioral (coefficients) changes was more important than that of the characteristic (endowments) changes in the decrement of thinness among late adolescent girls in Ethiopia in the last one and half-decade. Keeping the role of changes in compositional characteristics constant, about 84% of the decline in thinness among late adolescent girls in Ethiopia in the study period was attributed to a change in the effect of the characteristics (coefficient). Behavioral changes in nutrition among rural residents had a significant effect on the observed change in thinness. This finding is supported by evidence from a systematic- and meta-analysis in South and Southeast Asia (25), Dehradun district, India (26), Lay Gaynet district, Northwest Ethiopia (17), and Mizan district, Southwestern Ethiopia (27), where rural residents were undernourished than their counterparts and cultural and behavioral changes in feeding habits of rural society showed a positive effect in thinness reduction. Rural adolescents had poor access to safe water, and toilet facilities relatively lower educational attainment, and poor access and utilization of healthcare services than urban dwellers (28). Therefore, positive behavioral changes in rural adolescent girls might contribute to the decrement of thinness.

Another important factor that positively contributed to the decrement in thinness was marital status. Late adolescent girls who were not married significantly contributed to the decrement in thinness as compared with those who were married. The possible reason might be when adolescent girls get married before physical growth and developmental maturity, they will become pregnant with macro and micronutrient deficiencies or insufficiencies which in turn have negative nutritional and health status implications for them and their children (20).

After controlling the role of changes due to coefficients, 16% of the decrement in thinness among late adolescent girls in Ethiopia was due to differences in characteristics (endowments). Among the compositional factors; age, religion, and types of toilet had a statistically significant contribution to the change in thinness.

Regarding the age of adolescent girls, there was an increase in the proportion of adolescent girls who were 17 and 18 years old in the study period (Table 1) that positively attributed to the decrement in thinness. But the decrement in the composition of 19 years old adolescent girls in the sample showed a significant negative impact on the overall change in thinness. Similarly, the multilevel logistic regression analysis of this study indicated that the age of the adolescent girls had a statistically significant association with thinness. This finding is consistent with a study done among adolescent girls in Sub-Saharan Africa (29), a systematic review and meta-analysis done in Ethiopia (15), Mirab Armachiho District, Northwest Ethiopia (30), and another study in Lay Gaynet district, Northwest Ethiopia (17), where thinness was more likely in early-stage and slightly decreases in late adolescence. This could be because as compared to older adolescence, young adolescence is a period of growth spurt by which a physiological demand for macro and micronutrient demand is high. Besides younger adolescents are prone to erratic feeding habits and time for the autonomy of food choice. But if they cannot get adequate food at this age, their body mass index tends to decrease and they will become thin (31).

As a result of an increase in the proportion of protestant followers among the survey population in the study period, being protestant had a positive compositional contribution to the decrement of thinness among late adolescent girls in Ethiopia. This might be partially explained by most of the protestant followers in Ethiopia being urban dwellers, and being urban is less likely to be thin (27, 28, 32). However, there is no evidence whether religion affects the nutritional status of individuals, it needs further investigation.

The proportion of adolescent girls from households that had improved toilets declined in the study period (2000–2016). This change in the composition of the survey population resulted in a significant negative impact on the decrement of thinness. Similarly, the multilevel logistic regression analysis showed that late adolescent girls who were from households with unimproved toilets had higher odds to be thin. This finding is consistent with a systematic- and meta-analysis conducted in Ethiopia (15). Hence, this can be explained by an individual who had improper latrine utilization will have a higher risk for contaminations, intestinal parasites, communicable diseases, and infections which causes poor nutritional status (18).

This study revealed that the wealth status of the respondents had a statistically significant association with thinness. This finding is supported by evidence from South and Southeast Asia (25), Ghana (33), and Southern Ethiopia (34). This might be because adolescent girls from households with middle and low wealth index had lower purchasing power which leads to the consumption of suboptimal quality and quantity of food (35). As a result, late adolescent girls from households with a middle wealth index were more likely to be thinner.

In this study, late adolescent girls from female-headed households were less likely to be thin as compared to male-headed households. This might be explained by increasing a mother’s degree of autonomy at the household level which may impact the ability to make decisions in the best interest of the household members. When mothers have control over income, they tend to divert more toward health and nutrition-related expenditures than men. Therefore, empowering women and raising maternal autonomy is an important strategy for improving the nutritional status of children and adolescents to alleviate the global burden of malnutrition (36).

Residence by region showed a statistically significant association with thinness among late adolescent girls in Ethiopia from EDHS 2016. The odds of being thin were higher for late adolescent girls from the Somali region as compared to their counterparts from Addis Ababa. Moreover, late adolescent girls who were from Southern Nations Nationalities and people of the Ethiopian region had less odds of being thin as compared to late adolescents from Addis Ababa. This finding was consistent with a systematic review and meta-analysis study done in Ethiopia with a subgroup analysis by region which showed there was a high prevalence of thinness among adolescents in Eastern Ethiopia and lowest in Addis Ababa (15). This variety in adolescent thinness between regions could be due to recurrent drought, insufficient food production, household food insecurity differences, the difference in food culture, and genetic backgrounds, for example, Somali people as they are naturally taller and thinner with a minimal proportion of them are overweight and obese.

## Strength and Limitation

As a strength, this study was conducted based on the nationally representative population-based study that gives a high statistical power to infer the characteristics of the study population. Additionally, this study used a multivariate decomposition analysis to identify influential factors that could help policymakers to design interventions for decreasing thinness among late adolescent girls in Ethiopia. However, this study has also limitations. For example, the EDHS IR dataset lacks many nutritionally important factors, such as dietary diversity score (DDS), household food insecurity status, meal frequency, feeding habits, and others that affect nutritional status and it is indicated in the decomposition analysis with a large percent contribution of the constant.

## Conclusion

Thinness among late adolescent girls declined substantially, but it remains a high public health problem. The multivariate decomposition analysis revealed that more than 3/4th of the decrement in thinness among late adolescent girls in Ethiopia was attributed to the change in the effect of characteristics (coefficients) in the last one and half-decade. The place of residence and marital status were significantly associated with a change in thinness due to coefficients change. The compositional changes in the age of the adolescents, religion, and types of toilet facilities were also significantly associated with the change in thinness.

In this study, a multilevel logistic regression analysis identified a significant heterogeneity of thinness among clusters. From the fixed effect results, both individual and community-level factors had a significant association with thinness. Age of the adolescents, types of toilet facility, wealth index, and sex of head of the household were found significantly associated with thinness at an individual level, whereas being from Somali and Southern Nations Nationalities and people region had a statistically significant effect on the community level.

Therefore, we recommend to the government of Ethiopia, Ministry of Health, and Ministry of Education that nutrition programs and strategies targeting thinness reduction among late adolescent girls should base the identified factors like giving priority to rural and younger adolescents, enabling access and utilization of improved toilet facilities, improve the wealth status of households, empower women, and raise maternal autonomy through education. Regions identified with higher odds of thinness should be also prioritized for nutritional interventions. We recommend policymakers formulate and enforce laws and policies to prohibit the marriage of girls before 18 years of age. Furthermore, DHS lacks important nutritional variables like dietary diversity score (DDS), household food insecurity status, meal frequency, feeding habits, and others that affect the nutritional status of adolescents. So, we recommend the DHS program consider such variables for the next survey years. Besides, researchers need to further explore thinness from a religious perspective.

## Data availability statement

The datasets presented in this study can be found in online repositories. The names of the repository/repositories and accession number(s) can be found below: www.measuredhs.com.

## Author contributions

MZ, NM, and SN made the conceptualization, data curation, analysis, investigation, methodology, visualization, writing, review, and editing of the entire manuscript. All authors read and approved the final manuscript.

## Abbreviations

BAZ: body mass index for age
BMI: body mass index
DHS: demographic and health survey
EAs: enumeration areas
EDHS: Ethiopia Demographic and Health Survey
ICC: intra-class correlation coefficient
IRB: Institution Review Board
LMIC: lower- and middle-income countries
MOR: median odds ratio
PCV: proportional change in variance
SNNP: Southern Nations and Nationalities Region.
